# Comprehensive identification of cotton EPF/EPFL receptors and functional characterization of the GhEPFL1-1-GhER1 module in drought tolerance

**DOI:** 10.1186/s12870-025-06797-z

**Published:** 2025-07-11

**Authors:** Shichang He, Huijian Sun, Qing Chen, Yanlong Yang, Zixin Zhou, Saiwen Chang, Shuaiqi Lu, Zhencai Liang, Julan Yang, Xiao fei

**Affiliations:** 1 Xinjiang Key Laboratory of Biological Resources and Genetic Engineering, College of Life Science and Technology, Xinjiang University, Urumqi, 830046 China; 2 Bayingolin Mongolian Autonomous Prefecture Agricultural Science Research Institute, Korla, 841000 China; 3 Research Institute of Economic Crops, Xinjiang Academy of Agricultural Sciences, Urumqi, 830091 China

**Keywords:** EPF/EPFL, Gene family analysis, Cotton, Drought stress, *GhEPFL1-1*, *GhER1*, *GhSERK17*

## Abstract

The development of stomatal lineage cells in terrestrial plants is tightly regulated by epidermal patterning factors (EPFs/EPFLs) and their downstream receptors, including ERECTA, TOO MANY MOUTHS (TMM), and SOMATIC EMBRYOGENESIS RECEPTOR KINASEs (SERKs). These components form co-receptor complexes that activate the MAPK signaling cascade, playing critical roles in stomatal development, stress responses, and signal transduction. However, the EPF–ERECTA–TMM signaling network remains largely unexplored in cotton (*Gossypium* spp.). In this study, we performed a genome-wide identification and characterization of the *EPF/EPFL*, *ERECTA*, *TMM*, and *SERK* gene families in four cotton species (*G. hirsutum*, *G. barbadense*, *G. arboreum*, and *G. raimondii*), identifying 135 *EPF/EPFL*, 18 *ERECTA*, 6 *TMM*, and 90 *SERK* genes. Bioinformatics analyses—including gene collinearity, protein domain structure, cis-regulatory elements, and protein–protein interaction predictions—revealed functional divergence and stress-related regulatory potential across these families. Expression profiling in *G. hirsutum* indicated that several candidate genes, such as *GhEPFL1-1*, *GhER1*, and *GhSERK17*, are responsive to abiotic stresses. To validate these computational predictions, functional assays were conducted. Virus-induced gene silencing (VIGS) of *GhEPFL1-1*, *GhER1*, and *GhSERK17* led to increased stomatal density and reduced drought tolerance, confirming their roles in stress adaptation. Furthermore, luciferase complementation imaging in *Nicotiana benthamiana* demonstrated direct interactions between *GhEPFL1-1* and *GhER1*, and co-receptor complex formation with *GhSERK17*, consistent with molecular docking simulations. Collectively, this study lays a theoretical foundation for further exploration of the EPF/EPFL-mediated peptide-receptor signaling pathway in cotton and its potential application in breeding for stress resilience.

**Supplementary Information:**

The online version contains supplementary material available at 10.1186/s12870-025-06797-z.

## Introduction

Small peptides (SPs) are critical regulators of plant growth, development, and responses to environmental stresses [8, 39, 60]. Among them, the EPIDERMAL PATTERNING FACTOR (EPF) and EPF-LIKE (EPFL) peptides function as extracellular ligands that interact with receptor kinases to coordinate various developmental processes [33]. The *EPF/EPFL*-*ERECTA*, *TMM*, and *SERK* signaling pathways collectively regulate stomatal patterning, integument formation, and inflorescence stem elongation across diverse plant species [12, 33, 41, 47]. These peptides typically contain an N-terminal secretion signal and a C-terminal region characterized by six to eight conserved cysteine residues [43]. During protein maturation, the N-terminal signal peptide is cleaved, while the C-terminal cysteine residues facilitate disulfide bond formation, which is essential for their activation and biological function [43, 48].

In *Arabidopsis thaliana* (*A. thaliana*), the *EPF/EPFL* family comprises 11 members, including key regulators of stomatal development such as *EPF1*, *EPF2*, *CHALLAH/EPFL6*, and *STOMAGEN/EPFL9* [2, 14–16, 22, 23]. Their receptors, ERECTA (ER), ERL1, and ERL2, exhibit functional redundancy in controlling stomatal patterning [45, 46]. The ER family negatively regulates cell fate transitions and directs asymmetric cell division during stomatal development [45, 47]. Further studies have demonstrated that EPF/EPFL peptides modulate ER receptor activity through the ER-MAPK signaling cascade [20, 41, 58]. Overexpression of *AtEPF2* inhibits the differentiation of epidermal primary cells into meristemoid mother cells, thereby preventing excessive stomatal formation [15, 23]. Meanwhile, *AtEPF1* influences asymmetric cell division, ensuring optimal stomatal spacing and preventing clustering [14]. Both *AtEPF1* and *AtEPF2* are expressed in the epidermis, and their mutant and overexpression phenotypes confirm their role in activating ER family receptors [14, 22, 33].

In contrast, *EPFL9* (STOMAGEN), synthesized in mesophyll cells, promotes stomatal development by antagonizing *EPF1* and *EPF2*-mediated epidermal signaling [24, 32, 51, 62]. Overexpression of *EPF1*, *EPF2*, or *STOMAGEN* alters stomatal development in cotyledons and leaves when TMM function is intact, but these effects are abolished in *tmm-1* mutants [14, 15, 30, 51]. The receptor-like protein TMM facilitates heterodimerization of ER receptors and is indispensable for ER function in the cotyledon epidermis, as demonstrated by the phenotypic similarity between *tmm-1* mutants and *erl1 erl2* double mutants [33, 45]. Beyond stomatal regulation, ER signaling plays a broader role in plant development, including xylem and epidermal differentiation, where EPF/EPFL peptides act as upstream regulators [55]. Specifically, *EPFL4* and *EPFL6* redundantly control stem elongation and inflorescence growth by binding to ER receptors. The *epfl4 epfl6* double mutant exhibits severe dwarfism and compact inflorescences, whereas phloem-specific ER expression rescues elongation defects [7, 16, 55]. In addition to epidermal development, ER signaling regulates filament elongation, stamen development, and reproductive organ functionality in *A. thaliana* [2, 45, 53].

Receptor-like kinases (RLKs) frequently form multimeric receptor complexes, where ligand-induced transphosphorylation activates downstream signaling [13, 34]. The leucine-rich repeat II (LRRII) subfamily RLKs, including SERKs and CLAVATA3-insensitive receptor kinases (CIKs), serve as critical co-receptors that heterodimerize with distinct RLKs to regulate signal transduction [13, 19, 21, 26]. The *A. thaliana* genome encodes five *SERK* members: *SERK1*, *SERK2*, *SERK3*/*BAK1*, *SERK4*/*BKK1*, and *SERK5* [13, 17, 34]. The functional integrity of the EPF/EPFL-ERECTA module depends on SERK activity, as demonstrated by the epidermal and embryo sac developmental defects observed in *epfl1–epfl6* mutants and *erl1/erl2 serk1/2/3* multiple mutants [34]. Upon EPF-induced heterodimerization, ERECTA and SERK receptors undergo transphosphorylation, activating downstream pathways. Interestingly, SERK and TMM interact independently of ligand binding, and ERECTA, SERKs, and TMM assemble into multimeric receptor complexes to regulate stomatal patterning [34, 41].

Functional studies have demonstrated that the *EPF*/*EPFL-ERECTA* signaling pathway, in coordination with TMM and SERKs, plays a key role in plant adaptation to drought stress by modulating stomatal development [5, 11, 18]. This pathway enhances water use efficiency (WUE), thereby improving resilience to drought, heat, and other abiotic stresses. Heterologous overexpression of *HvEPF*1, *OsEPF1*, and *OsEPF2* in *A. thaliana* significantly reduces stomatal density, confirming the conserved function of EPF peptides in optimizing water conservation [38]. Similar regulatory mechanisms have been observed in monocots: overexpression of *OsEPFL9* in rice (*Oryza sativa*) and *TaEPF1B* in wheat (*Triticum aestivum*) alters stomatal density and WUE, directly correlating with enhanced drought tolerance [25, 44, 61]. Drought-responsive modulation of *EPF* homologs extends to other crops, including *Solanum tuberosum* (potato) and *Sorghum bicolor* [27, 29, 36]. Under drought stress, *EPF* genes in maize and rye also exhibit significant expression changes [35, 59, 66]. *EPFL1-6* is sensed by the ERECTA-SERK complex to control epidermal development in *A. thaliana*, affecting stomatal patterning and plant water-use strategies [11, 18, 34].

As a globally significant cash crop, cotton (*Gossypium* spp.) is highly susceptible to abiotic stresses such as drought, high temperature, and soil salinity, which severely impact yield. While the EPF/EPFL-ERECTA/TMM/SERK signaling module has been extensively characterized in model plants (*A. thaliana*), its regulatory functions in cotton remain largely unexplored. To address this gap, we conducted a genome-wide identification of EPF/EPFL peptide hormones and their cognate receptors (ER, TMM, and SERKs) in four cotton species: *G. hirsutum* (upland cotton), *G. barbadense* (sea island cotton), *G. arboreum*, and *G. raimondii*. Comprehensive bioinformatics analyses were performed, including chromosomal localization, upstream cis-acting element identification, collinearity assessment, and protein characterization. Expression patterns under drought and salt stress were examined using transcriptomic data and validated via quantitative reverse transcription PCR (RT-qPCR). Notably, RT-qPCR analysis revealed significant upregulation of key pathway genes under drought and salt stress. To further elucidate the functional mechanisms underlying drought tolerance.Three candidate genes (*GhEPFL1-1*, *GhER1*, and *GhSERK17*) were prioritized for functional validation through a systematic evaluation of their responsiveness to drought stress and phylogenetic correlation with *Arabidopsis thaliana* homologs (*AtERECTA* and *AtSERK3*). we employed virus-induced gene silencing (VIGS) targeting *GhEPFL1-1*, *GhER1*, and *GhSERK17* in cotton, virus-induced gene silencing (VIGS) of GhEPFL1-1, GhER1, and the co-receptor GhSERK17 significantly increased stomatal density and reduced enzymatic antioxidant activities and chlorophyll content, ultimately impairing drought resistance in cotton. Additionally, molecular interactions were validated using dual-luciferase (LUC) reporter assays in *N. benthamiana*.

This study provides new insights into the EPF/EPFL–ERECTA/TMM/SERK signaling pathway in cotton, offering a foundation for future applications in drought-resilient breeding. Notably, it represents the first systematic genome-wide identification and functional validation of EPF/EPFL peptides and their receptors in *Gossypium* species. In contrast to previous studies that were largely limited to in silico predictions, our work integrates comprehensive bioinformatics analysis with experimental approaches—including virus-induced gene silencing (VIGS), luciferase complementation imaging (LCI), and molecular docking—to confirm the functional relevance of the GhEPFL1-1–GhER1–GhSERK17 module. This integrative strategy establishes a mechanistic framework for understanding peptide-receptor signaling in cotton and highlights novel targets for improving drought tolerance through molecular breeding.

## Materials and methods

### Plant materials and stress treatments

For this study, *G. hirsutum* TM-1 was selected as the experimental material. Cotton tissues, including roots, stems, leaves, flowers and fibers, were cultivated in a controlled plant growth chamber. Prior to sowing, seeds were soaked in double-distilled water (ddH₂O) for 3 to 5 h and then planted in sandy soil. Plants were grown under greenhouse conditions until the three-leaf stage, with a photoperiod of 14 h light (28–30 °C) and 10 h dark (25–28 °C) at a light intensity of 150 µmol m⁻^2^ s⁻^1^. For stress treatments, seedlings were divided into three experimental groups and a control group. The control group was treated with ddH₂O at 25 °C for 24 h. The drought stress group was subjected to 18% polyethylene glycol (PEG) 6000 solution at 25 °C for 24 h to simulate osmotic stress. The salt stress group was treated with 150 mM NaCl solution at 25 °C for 24 h. Each treatment was performed in triplicate, with 15 plants per biological replicate. Leaf samples were collected at 1,3,6, 12, and 24 h post-treatment, immediately frozen in liquid nitrogen, and stored at − 80 °C for subsequent RNA extraction.

### Identification of EPF/EPFL, ERECTA, TMM, and SERK family members in cotton

The protein sequences of the three *ERECTA* homologs, one *TMM*, and five *SERK* genes in *A. thaliana* were retrieved from the TAIR database (https://www.arabidopsis.org/). Genomic assembly sequences for *G. arboreum* (CRI), *G. raimondii* (JGI), *G. barbadense* (HAU), and *G. hirsutum* (CRI) were obtained from the Cotton Functional Genomics Database (https://cottonfgd.org). To identify candidate gene family members, homologs of *A. thaliana* ERECTA, TMM, and SERK proteins were systematically identified in *G. hirsutum* using BLASTP searches against the cotton protein database, applying an E-value cutoff of ≤ 1 × 10⁻^15^ and a sequence identity threshold of ≥ 50%. All protein sequences meeting these criteria were retained. For each gene, only the primary transcript isoform was selected for further characterization to avoid redundancy (Supplementary Table 1). Putative homologs were further validated through domain analysis using Pfam (http://pfam.xfam.org/scan) and the NCBI Conserved Domain Database (http://www.ncbi.nlm.nih. gov/cdd/). Proteins were selected for further analysis only if they contained the characteristic domains of their respective families. Specifically, ERECTA and SERK proteins were required to contain both leucine-rich repeat (LRR) and kinase domains, while TMM proteins were required to possess LRR domains.

### Multiple sequence alignment, phylogenetic analysis, and phylogenetic tree construction

Multiple sequence alignment of EPF/EPFL, ERECTA, TMM, and SERK protein sequences from four *Gossypium* species and *A. thaliana* was performed using DNAMAN 7.0. The aligned sequences were then imported into MEGA7 for downstream analysis [50]. To assess the potential impact of alignment quality, we evaluated the effect of trimming low-confidence regions. Comparative analysis showed that alignment trimming did not significantly affect branch support values or tree topology across gene families. Therefore, full-length alignments were retained for phylogenetic reconstruction to preserve informative sequence variation. The best-fit substitution model was determined using ProtTest 3, which selected the JTT + Γ + I model under both AIC and BIC criteria. Phylogenetic trees were constructed using IQ-TREE with the JTT + Γ + I model, and branch support was estimated with 1000 ultrafast bootstrap replicates. The resulting trees were visualized as majority-rule consensus trees, displaying branch lengths from the maximum-likelihood best tree and UFBoot support values at internal nodes. Unless otherwise stated, all alignment and tree construction parameters were set to default values to ensure reproducibility.

### Conserved domain and motif analysis

Conserved motifs in EPF/EPFL, ERECTA, TMM, and SERK proteins from cotton were identified using the MEME Suite (https://meme-suite.org/meme/) with search parameters set to detect motifs ranging from 25 to 200 amino acids in length, with a maximum of 10 motifs per sequence. Multiple sequence alignment of these protein families was conducted using Clustal Omega (https://www.ebi.ac.uk/Tools/msa/clustalo/). Gene structure analysis, including intron–exon organization, was performed using TBtools [9]. Additionally, TBtools was used to integrate evolutionary relationships, gene structures, and conserved motifs of the EPF/EPFL, ERECTA, TMM, and SERK families into a comprehensive visualization.

### Analysis of physicochemical properties, secondary structure, and intra-/extracellular domains

The physicochemical properties of ERECTA, TMM, and SERK proteins, including molecular weight, theoretical isoelectric point (pI), instability index, aliphatic index, and grand average of hydropathicity (GRAVY), were analyzed using ExPASy ProtParam (https://web.expasy.org/ protparam/) [4]. Secondary structure predictions, including α-helices, β-sheets, random coils, and extended strands, were conducted using PSIPRED v4.0 (http://bioinf.cs.ucl.ac.uk/psipred/) for fold recognition and SOPMA (https://npsa-prabi.ibcp.fr/cgi-bin/npsa automat.pl?page =/NPSA/npsa sopma.html) for consensus secondary structure composition. Signal peptides, transmembrane domains, and intracellular/extracellular regions were identified using SignalP-5.0 (https://services.healthtech.dtu.dk/service.php?SignalP-5.0) with default thresholds. To ensure prediction accuracy, all algorithms were validated against the UniProtKB/Swiss-Prot reference database [3].

### Chromosomal localization, gene duplication, and homology analysis

The chromosomal positions of *EPF/EPFL*, *ERECTA*, *TMM*, and *SERK* genes were retrieved from gene annotation files of the four cotton species. These genes were then mapped onto their respective chromosomes using TBtools. Gene duplication events were identified using the Multicollinearity Scanning Toolkit (MCScanX) [56]. Synonymous (Ks) and non-synonymous (Ka) substitution rates for *EPF/EPFL* gene pairs were calculated using the Ka/Ks calculator in TBtools v1.051 (https://github.com/CJ-Chen/TBtools) [9].

### Analysis of cis-acting regulatory elements, microRNA prediction, and gene expression patterns

Promoter regions (2 kb upstream of transcription start sites) of *ERECTA*, *TMM*, and *SERK* genes in cotton were extracted using custom Perl scripts. Cis-regulatory elements within these promoter sequences were identified using the PlantCARE database (http://bioinformatics.psb. ugent.be/webtools/plantcare/html/) and visualized with TBtools [40]. Tissue-specific and stress-responsive expression profiles of *GhEPF/EPFL*, *TMM*, *ERECTA*, and *SERK* genes were analyzed using publicly available RNA-seq data from the COTTONOMICS database (https://cottonomics.zju.edu.cn/). The dataset comprised samples from various developmental stages (e.g., ovules and fibers), multiple plant organs, and abiotic stress conditions (cold, heat, drought, and salt) over treatment durations ranging from 1 to 24 h. Gene expression heatmaps were generated using TBtools based on normalized FPKM (fragments per kilobase of transcript per million mapped reads) values, which were log₂-transformed and Z-score normalized to facilitate visualization and comparative analysis. MicroRNA (miRNA) target prediction was performed by querying the coding sequences of target genes against the psRNATarget database (http://plantgrn.noble.org/psRNA Target/) using default parameters (Dai et al., 2018). Prediction results were filtered using a maximum expectation score threshold of ≤ 5.0, while all other parameters were retained at their default settings as specified by the tool.

### Expression analysis of *GhEPF/EPFL*, *GhTMM*, *GhERECTA*, and *GhSERK *Genes in *G. hirsutum*

The expression patterns of *GhEPF/EPFL*, *TMM*, *ERECTA*, and *SERK* genes in *Gossypium hirsutum* were analyzed across different tissues and under various abiotic stress conditions. Total RNA was extracted using the EASYspin Plus Plant RNA Kit (Aidlab, Beijing, China) following the manufacturer's protocol. First-strand complementary DNA (cDNA) was synthesized using the PrimeScript RT Reagent Kit (Perfect Real Time, Takara, Dalian, China). The cDNA samples were diluted five-fold with double-distilled water (ddH₂O) for quantitative real-time PCR (qRT-PCR) analysis using the ABI 7500 Fast Real-Time PCR System. Primers (Supplementary Table 2) were designed using the GenScript online tool (https://www.genscript.com/tools/real-time-pcr-taqman-primer-design-tool) based on standard primer design principles, and their specificity was validated using Primer-BLAST in NCBI (https://www.ncbi.nlm.nih.gov/tools/primer-blast/index.cgi). *GhActin* (AY305733) was used as the reference gene, and the 2^⁻ΔΔ^CT method was applied to calculate the relative expression levels of *GhEPF/EPFL*, *TMM*, *ERECTA*, and *SERK* genes in each sample. Expression analysis was conducted under stress conditions using three biological replicates.

### Silencing of *GhEPF/EPFL* Genes in cotton and determination of physiological parameters related to drought stress

Fragments of *GhEPFL1-1*, *GhER1*, and *GhSERK17* were cloned into the *pYL156* vector, while the empty vector *pYL156* and *pYL156:CLA1* were used as negative and positive controls, respectively. The recombinant plasmids were introduced into *Agrobacterium tumefaciens* strain GV3101. The *pYL156*, *pYL156*:*CLA1*, and *pYL156* constructs containing *GhEPFL1-1*, *GhER1*, and *GhSERK17* were mixed in equal proportions with the auxiliary vector *pYL192*. The resulting bacterial suspension was infiltrated into the cotyledons of seven-day-old TM-1 seedlings. The efficiency of gene silencing in virus-induced gene silencing (VIGS) plants was evaluated by qRT-PCR. For drought stress treatment, three-leaf stage seedlings were subjected to 18% polyethylene glycol (PEG) 6000 treatment to simulate drought conditions. Leaf samples were collected 24 h after PEG-induced drought treatment. Each treatment was performed with three independent biological replicates. Proline (Pro) content was measured using 0.1 g of cotton leaf tissue with the Pro Quantification Assay Kit (Nanjing Jiancheng). Hydrogen peroxide (H₂O₂) content was determined with the corresponding H₂O₂ Quantification Assay Kit. The activities of superoxide dismutase (SOD), peroxidase (POD), and catalase (CAT) enzymes were quantified in both treated and control samples using commercially available assay kits from the same manufacturer. Chlorophyll was extracted with 96% (v/v) ethanol, and absorbance was measured at 649 nm and 665 nm. Chlorophyll content was calculated using the formula: Chl = (13.95 × A665 − 6.88 × A649) + (24.96 × A649 − 7.32 × A665) × (extraction volume/sample fresh weight). Statistical analysis of physiological assay results was conducted using one-way analysis of variance (ANOVA), followed by Tukey’s HSD test for multiple comparisons. Statistical significance was set at *P* < 0.05. All data are presented as mean ± standard deviation (SD).

#### Luciferase complementation imaging assay

*N. benthamiana* plants were cultivated in a growth chamber at 25 °C with a 16 h light/8 h dark photoperiod, using a soil-to-perlite ratio of 3:1 as the growth medium. These plants were used for the split luciferase complementation assay. The full-length coding sequences of *GhEPFL1-1*, *GhER1*, and *GhSERK17* were cloned into *pCAMBIA1300*-nLUC (encoding the N-terminal half of LUC) and *pCAMBIA1300*-cLUC (encoding the C-terminal half of LUC), respectively. The recombinant plasmids were individually introduced into *Agrobacterium tumefaciens* strain GV3101. Positive transformants were cultured overnight in LB medium, resuspended in MS medium to an OD₆₀₀ of 0.9, mixed at a 1:1 ratio (v/v), and co-infiltrated into the leaves of four-week-old *N. benthamiana* plants. The infiltrated plants were incubated in darkness for one day, followed by a 24-h light period. The Stable-Lite Luciferase Assay System (Vazyme, Nanjing, China) was used for protein extraction and LUC luminescence reactions. Luciferase activity was detected using a ChemiDoc MP Imaging System (Bio-Rad, Hercules, USA) to assess interaction strength. Negative controls included combinations of empty nLUC/cLUC vectors with cLUC*-ER*, nLUC*-GhEPFL1-1*, or nLUC*-GhSERK17*.

#### Molecular docking model prediction

For molecular docking predictions, the protein sequences of GhEPFL1-1, GhER1, and GhSERK17 were uploaded to SWISS-MODEL (https://swissmodel.expasy.org/) to obtain PDB files with the highest sequence similarity and coverage [57]. The downloaded PDB files were then submitted to GRAMM (https://gramm.compbio.ku.edu/request) for protein–protein docking model generation [54]. The resulting models were analyzed using PDBePISA (https://www.ebi.ac.uk/pdbe/pisa/) to predict docking interactions and identify amino acid residues involved in hydrogen bonding.

## Results

### Identification and characterization of EPF/EPFL, ERECTA, TMM, and SERK family members in cotton species

**Fig. 1:**
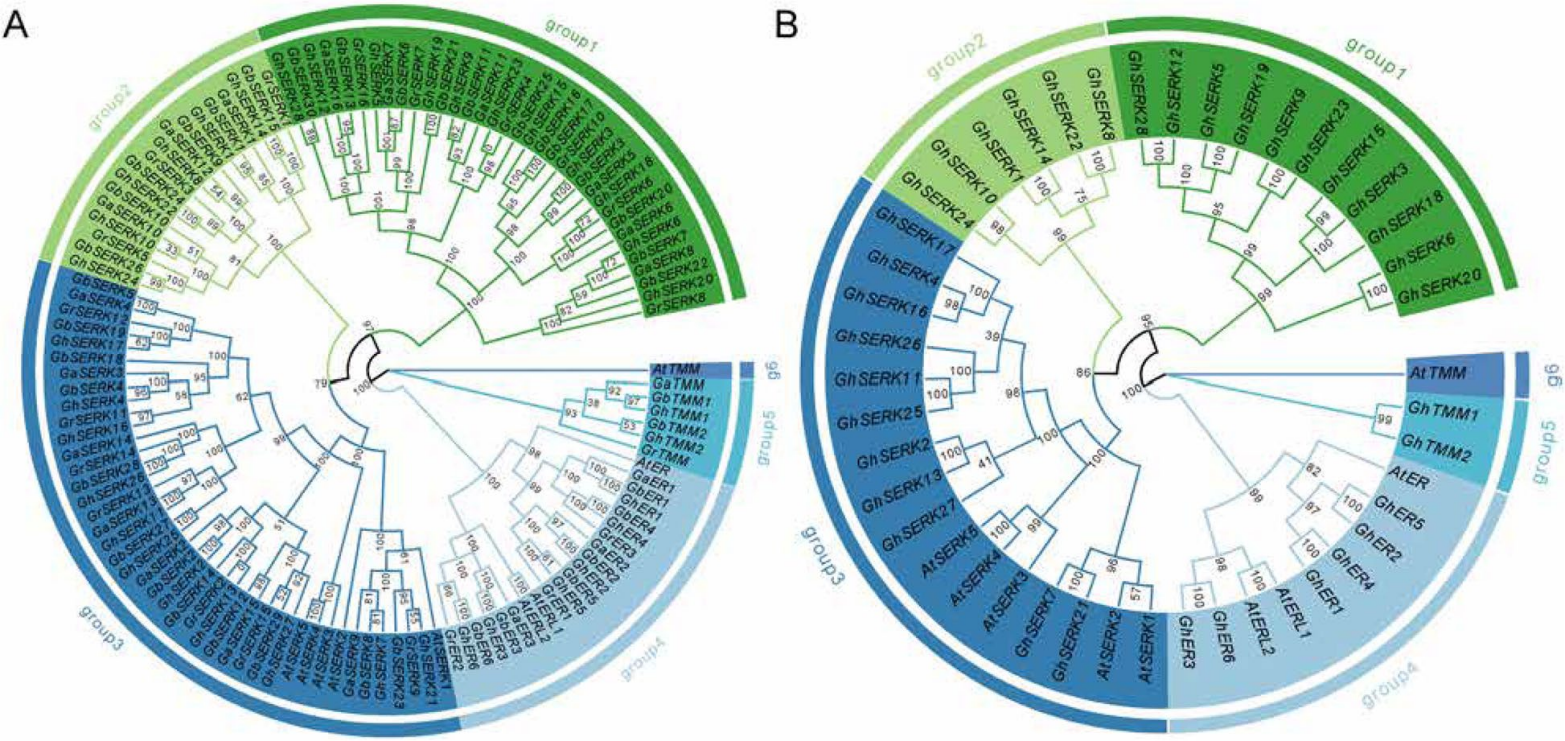
Phylogenetic analysis of *ER*, *TMM*, and *SERK* genes in *A. thaliana*, *G. arboreum*, *G. raimondii*, *G. hirsutum*, and *G. barbadense*. **A** Phylogenetic tree of *ER*, *TMM*, and *SERK* genes in *A. thaliana* (At), *G. arboreum* (Ga), *G. raimondii* (Gr), *G. hirsutum* (Gh), and *G. barbadense* (Gb). **B** Phylogenetic tree of *ER*, *TMM*, and *SERK* genes in *Arabidopsis thaliana* (At) and *Gossypium hirsutum* (Gh). Bootstrap support values are shown at internal branch nodes and indicate the statistical confidence of each inferred clade

A total of 45, 44, 23, and 23 putative *EPF/EPFL* genes were identified in *Gossypium hirsutum*, *G. barbadense*, *G. arboreum*, and *G. raimondii*, respectively (Fig. S1 A–B). Subsequent analyses focused on the *ERECTA* (ER), *TMM*, and *SERK* gene families. Gene copy number was positively correlated with genome ploidy: the tetraploid species (*G. hirsutum* and *G. barbadense*) possessed six *ER* genes and two *TMM* genes, whereas diploid species (*G. arboreum* and *G. raimondii*) harbored three *ER* and one *TMM* gene. Chromosomal mapping revealed conserved synteny across species: *ER* genes were located on chromosomes 6, 7, and 11 in diploids and on homoeologous chromosomes A06/D06, A07/D07, and A11/D11 in tetraploids. *TMM* genes were generally mapped to chromosome 5 (Chr5, A05, D05), except for *G. raimondii*, in which *TMM* localized to chromosome 9 (Fig. S2). The *SERK* gene family showed considerable expansion in tetraploid species (*G. hirsutum*: 28 genes; *G. barbadense*: 30 genes) compared to diploids (16 genes each in *G. arboreum* and *G. raimondii*), with up to four members per chromosome, suggesting lineage-specific duplication. Maximum likelihood phylogenetic analysis resolved *ERECTA*, *TMM*, and *SERK* gene families into six distinct subgroups using *Arabidopsis* homologs as reference (Fig. 1A). *TMM* and *ERECTA* formed well-supported monophyletic clades, while *SERK* genes segregated into three major subgroups. Bootstrap support values for ancestral nodes of orthologous groups across all five species exceeded 79, indicating statistically reliable groupings. Within the *TMM* and *ERECTA* clades, bootstrap values approached 100%, underscoring strong phylogenetic support. Notably, the phylogenetic topologies of *G. hirsutum* and *A. thaliana* mirrored each other, with all three gene families clustering into the same six subgroups. Bootstrap values for ancestral nodes of orthologous clades in this comparison consistently exceeded 86, further validating evolutionary conservation. Within the *ERECTA* family, *GhER3* and *GhER6* clustered with *AtERL1* and *AtER2*, while the remaining *GhER* members grouped with *AtER1*. In the *SERK* family, subgroup III included *GhSERK* genes most closely related to *AtSERK* homologs, suggesting potential functional conservation based on both phylogenetic proximity and domain structure (Fig. 1B).

### Analysis of physicochemical properties, secondary structure, and extracellular and intracellular domains of *ERECTA*, *TMM*, and *SERK* in cotton

A comprehensive analysis of the physicochemical properties of TMM, ERECTA, and SERK proteins revealed distinct characteristics among these receptor families (Supplementary Table 3). Members of the TMM family displayed highly conserved features, with consistent amino acid lengths (489 aa), molecular weights (53.4–53.8 kDa), alkaline isoelectric points (pI: 8.02–8.29), moderate instability indices (34.29–35.76), relatively low aliphatic indices (91.9–93.5), and slight hydrophilicity (GRAVY: –0.088 to –0.026). In contrast, ERECTA proteins were substantially larger (944–993 aa; MW: 104–110 kDa), exhibited near-neutral pI values (5.82–6.31), comparable instability indices (33.9–35.4), higher aliphatic indices (104.46–107.18), and near-neutral GRAVY scores (–0.051 to –0.02), indicating greater thermal stability and solubility relative to TMM proteins.

SERK proteins showed the greatest variability in length and physicochemical traits, ranging from 452 to 673 aa and 50 to 74 kDa in molecular weight. Their isoelectric points varied widely (5.24–8.51), and instability indices ranged from 28.6 to 43.8 (e.g., GaSERK12: 32.28; GbSERK25: 43.79). GRAVY values were consistently negative (–0.242 to –0.006), suggesting a general tendency toward hydrophilicity. Secondary structure predictions revealed that α-helices (32.65–43.56%), extended strands (6.75–13.6%), and random coils (49.69–57.93%) were the predominant structural elements across these proteins (Supplementary Table 4). Among them, TMM proteins exhibited the highest α-helix content (up to 43.56%), implying structural rigidity, whereas SERK proteins contained more random coil regions (up to 57.93%), suggesting greater structural flexibility. No β-turns were predicted, likely due to limitations in the secondary structure prediction algorithm. All members of these gene families exhibited negative GRAVY scores and elevated aliphatic indices, indicating moderate hydrophilicity and a tendency toward increased structural stability. These physicochemical properties suggest that the encoded proteins may remain functionally stable under stress conditions such as drought. In particular, high aliphatic index values are associated with enhanced thermal stability, while negative GRAVY scores may facilitate protein solubility and interaction with aqueous cellular environments. Together, these features may contribute to osmotic homeostasis and protein resilience during drought adaptation.

Subcellular domain analyses demonstrated conserved yet distinct domain architectures among these proteins. TMM proteins possessed a conserved signal peptide region (1–22 aa), followed by an extensive extracellular domain (22–489 aa). ERECTA proteins featured longer signal peptides (21–33 aa), larger extracellular domains (532–562 aa), shorter transmembrane helices (19–21 aa), and substantial intracellular domains (368–382 aa). In contrast, SERK proteins showed variability in signal peptide length (19–28 aa), moderately sized extracellular domains (197–209 aa), and prominent intracellular domains (224–390 aa) (Supplementary Table 5). Notably, the observed length variations in the extracellular domains of ERECTA and SERK proteins suggest potential adaptive divergence associated with recognition specificity of EPF/EPFL ligands, critical components involved in stress signaling in plants.

### Motif and structural analysis of ERECTA, TMM, and SERK proteins

**Fig. 2:**
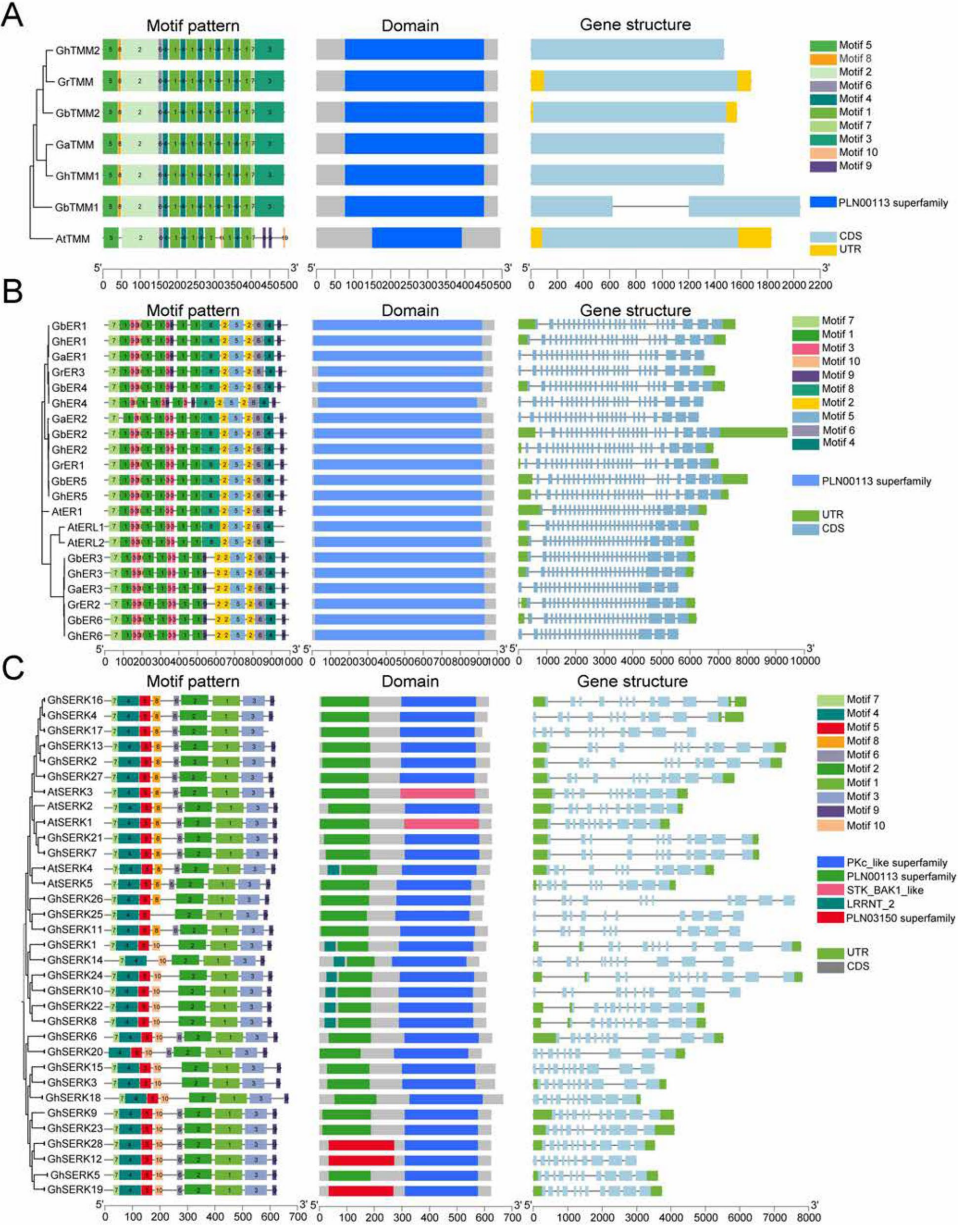
Phylogenetic relationships, motif compositions, gene domains, and gene structures of *ERECTA*, *TMM*, and *SERK* genes in *A. thaliana*, *G. arboreum*, *G. raimondii*, *G. hirsutum*, and *G. barbadense*. **A** *TMM* genes. **B** *ERECTA* genes. **C** *SERK* genes (*A. thaliana* and *G. hirsutum* only)

Integrated analyses of phylogenetic relationships, gene structures, and conserved motifs revealed characteristic structural features shared among the ERECTA, TMM, and SERK receptor protein families in cotton (Fig. 2). All TMM proteins harbored a single conserved PLN00113 superfamily domain. Gene structure analysis revealed limited variation in intron–exon organization among TMM homologs. For example, *GrTMM*, *GbTMM2*, and *AtTMM* contain two annotated untranslated regions (UTRs), whereas other *TMM* genes lack clearly defined UTRs. Most *TMM* genes possess a single coding sequence (CDS), with the exception of *GbTMM1*, which contains two annotated CDS regions. Interestingly, despite this structural deviation, *GbTMM1* exhibits conserved motif composition and arrangement, suggesting that the observed variation may not substantially alter its splicing behavior or protein architecture. MEME motif analysis showed high conservation across cotton TMM homologs, exhibiting uniform motif numbers (8–9 motifs) and consistent motif arrangements (Fig. 2A). However, AtTMM notably differed in motif arrangement, characterized by the absence of motif 3 and the presence of an additional motif 9 relative to cotton orthologs (Fig. S3).

ERECTA family genes exhibited notable variation in exon–intron structure, with most members containing 26 or 27 exons and 0–2 annotated untranslated regions (UTRs). Proteins encoded by genes with 26 exons showed distinct motif architectures compared to those with 27 exons. Specifically, motif 8 was present in the 26-exon group, whereas motifs 2 and 9 were observed in the 27-exon group, suggesting that exon number variation may contribute to divergence in conserved motif composition (Fig. 2B). Despite these genomic structural differences, all ERECTA proteins retained the conserved PLN00113 domain, a hallmark of the LRR-RLK receptor-like kinase superfamily. MEME analysis identified ten conserved motifs across the ERECTA family, with subgroup-specific distribution patterns. Notably, motif 1 appeared in up to five copies, motif 3 in three to four copies, and motif 2 in two to three copies, whereas motifs 4 through 10 were each present in single copies per sequence (Fig. S4).

SERK family genes displayed highly conserved exon–intron organization across most homologs, typically comprising 11 exons and two UTRs in both *G. hirsutum* and *Arabidopsis*. MEME analysis revealed nine conserved motifs (motifs 1–7 and 9–10) consistently shared among SERK proteins from multiple species, while motif 8 showed lineage-specific presence or absence (Fig. 2C). *G. raimondii* and *G. barbadense* SERK proteins exhibited nearly identical motif arrangements; however, some homologs (e.g., GrSERK9) contained additional unique motifs, suggesting possible lineage-specific functional diversification (Figs. S5–S7). The typical domain architecture of SERK proteins includes an N-terminal signal peptide, followed by 4.5 to 5 leucine-rich repeats (LRRs), corresponding to motifs 4, 5, and 7. This is succeeded by an intracellular serine/threonine kinase domain (STK_BAK1_like), represented by motifs 1, 2, and 3, and a conserved C-terminal region [1]. Additionally, the Ser–Pro–Pro (SPP) signature sequence characteristic of this family is reflected in motifs 8 and 10. MEME analysis further confirmed that these structural features are consistently conserved across all identified SERK members (Supplementary Table 6). To support the reliability of motif prediction, E-values for all identified motifs are provided in Supplementary Table 7.

### Evolutionary analysis of ERECTA, TMM, and SERK in four *Gossypium* species

**Fig. 3:**
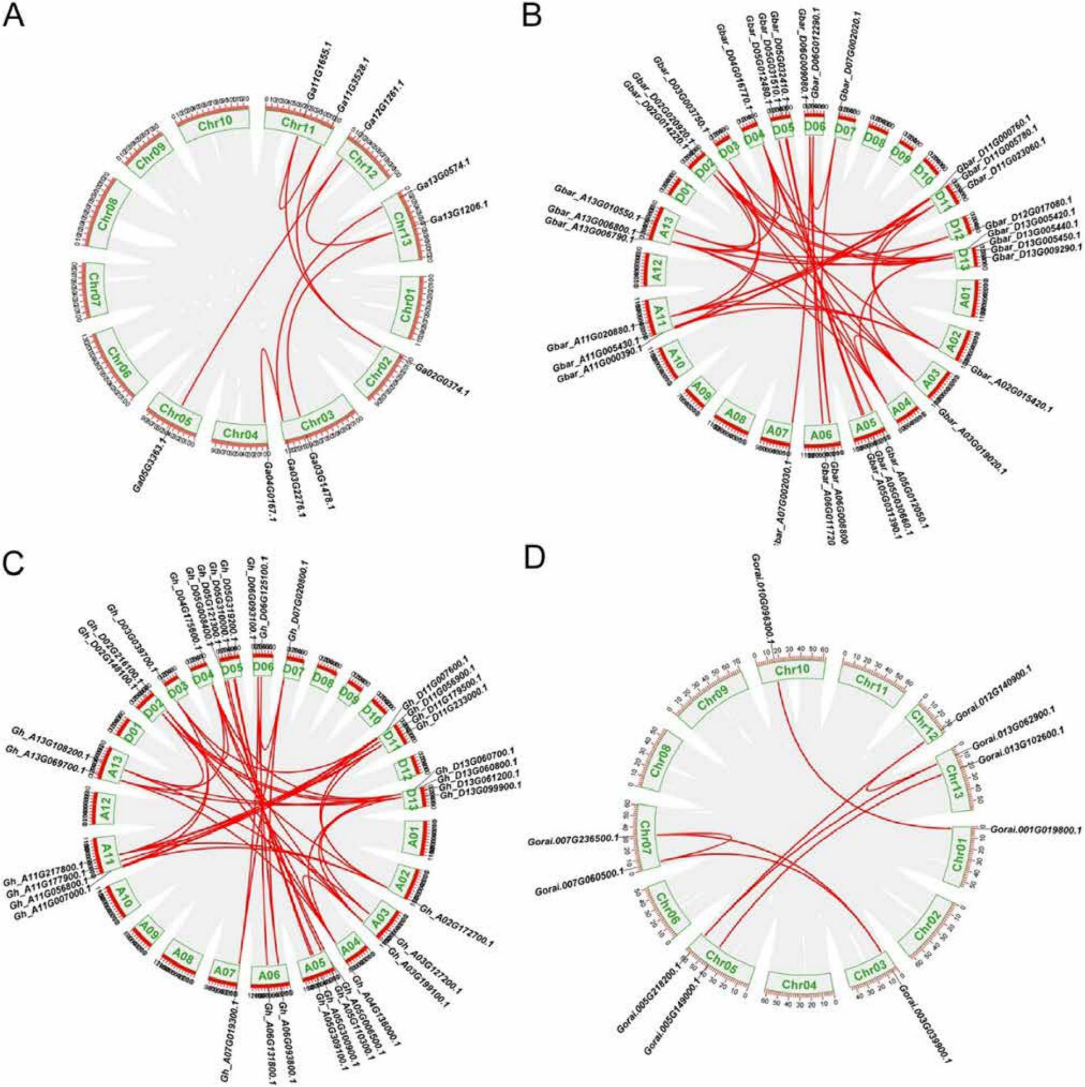
Distribution of ER, TMM, SERK homologous pairs on chromosomes in intra-genomics of four cotton species. A G. arboreum; B G. raimondii; C G. barbadense; D G. hirsutum

**Fig. 4:**
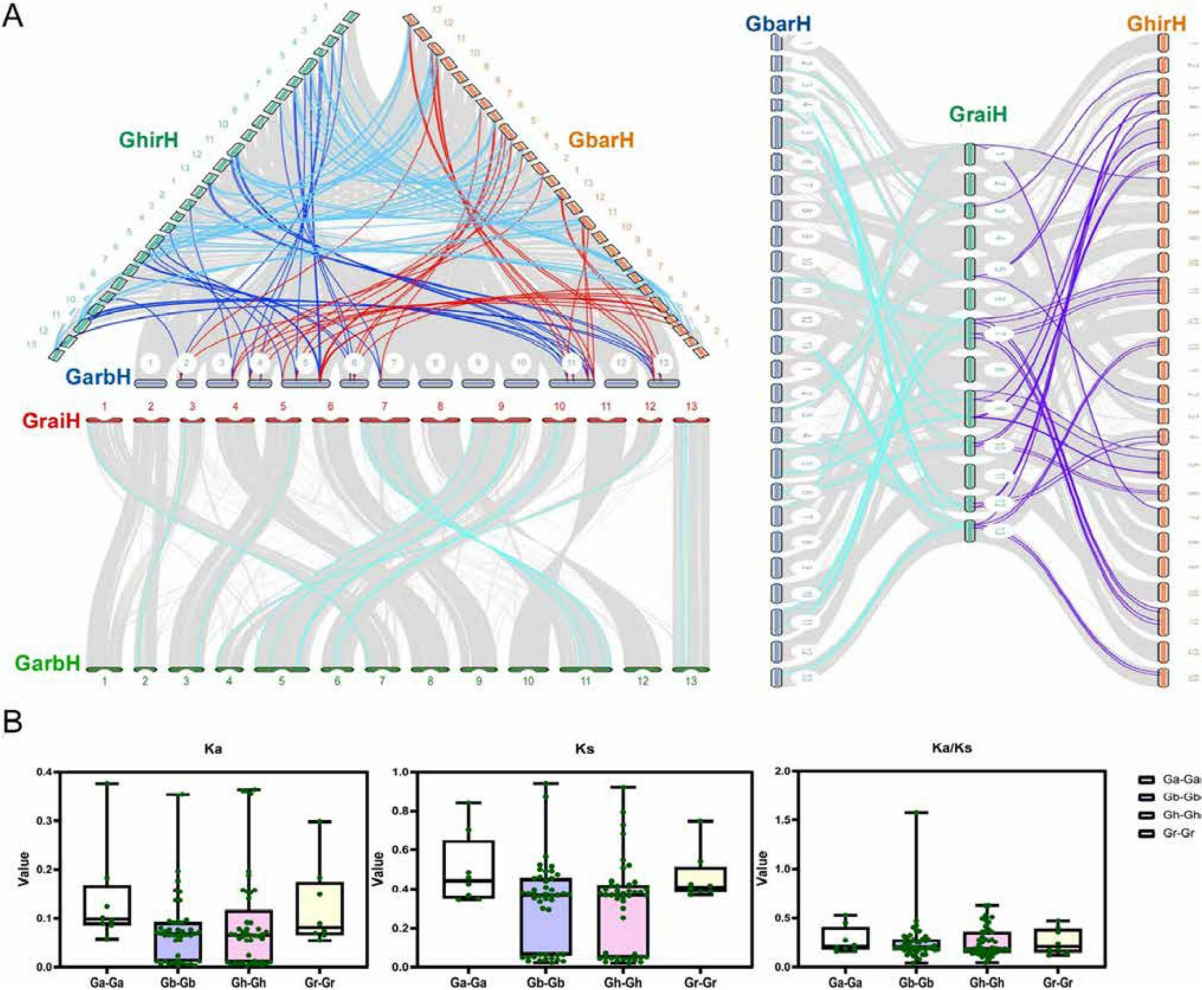
Distribution of *ER* *,* *TMM* *,* *SERK* homologous pairs on chromosomes in intra-genomics of four cotton species. **A** The collinear relationship betweenn *G. arboreum and G. hirsutum* *,* *G. arboreum and G. barbadense* *,* *G. raimondii and G. barbadense* *,* *G. raimondii and G. hirsutum* *,* *G. barbadense and G. hirsutum*. The gray lines: the collinearity of the whole genome among cotton species. The colorful lines: the collinearity of ER, TMM, SERK pairs in inter genomics. **B** Analysis of Ka/Ks. The green spot denote the Ka/Ks ratios, Ka values, and Ks values, respectively, of the syntenic gene pairs

To elucidate the evolutionary origins and selective constraints of the *ERECTA*, *TMM*, and *SERK* gene families in cotton, intra- and inter-species collinearity analyses were conducted across four representative *Gossypium* species (Figs. 3A–D). The intra-species analysis identified one syntenic pair of the *TMM* gene within each tetraploid species (*G. hirsutum* and *G. barbadense*). In contrast, ERECTA and SERK homologs displayed distinct subgenome-specific duplication patterns, exhibiting a notably higher number of syntenic pairs (31–32 A-D subgenome pairs in tetraploids) compared to diploid species (6–8 A-A or D-D pairs). Inter-species comparisons revealed extensive conserved syntenic blocks, notably with 59 homologous pairs identified between the two tetraploid species, *G. hirsutum* and *G. barbadense*, underscoring their recent common polyploid origin (Fig. 4A). Ka/Ks ratio analysis indicated strong purifying selection across all three gene families, suggesting functional conservation of *ERECTA*, *TMM*, and *SERK* genes throughout cotton evolution (Fig. 4B). Collectively, these findings imply that purifying selection pressures have conserved the ancestral roles of these receptor kinases in stress responses and developmental regulation in *Gossypium* species.

### Analysis of cis-acting regulatory elements of *ERECTA*, *TMM*, and *SERK* genes in cotton

**Fig. 5:**
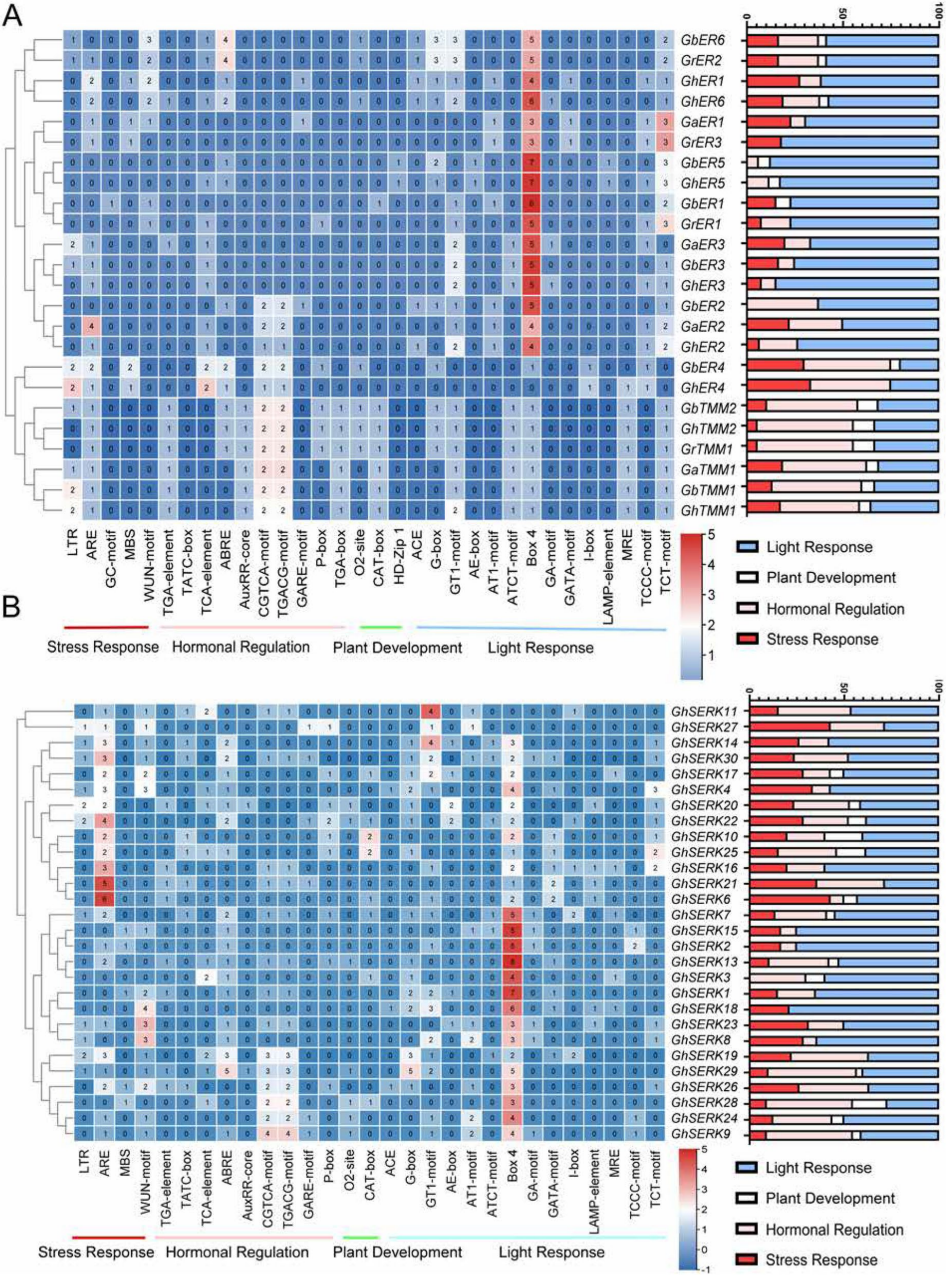
Analysis of cis-acting element numbers in TMM, ERECTA, SERK genes. **A** The colorful level of the grid indicate the number of cis-acting elements of the ER and TMM in *G. hirsutum, G. arboreum*, *G. raimondii* and *G. barbadense*. **B** The colorful level of the grid indicates the number of cis-acting elements of the *GhSERKs*

To elucidate the potential regulatory mechanisms governing the expression of *ERECTA*, *TMM*, and *SERK* genes, cis-acting elements within 2-kb promoter regions upstream of their transcription start sites were analyzed using the PlantCARE database. The analysis identified a diverse array of cis-elements, predominantly associated with light responsiveness and hormone signaling. In all four cotton species examined, light-responsive elements—particularly Box 4 motifs—were the most abundant among the *ERECTA*, *TMM*, and *SERK* gene promoters (Figs. 5A, B). These elements suggest a potential role in photomorphogenic regulation. Additionally, hormone-responsive elements were prevalent, including the CGTCA- and TGACG-motifs (key components of the jasmonic acid (JA) signaling pathway), the TGA-element (involved in auxin response), and ABRE (the abscisic acid (ABA) responsive element). These hormone-related elements are known to participate in stomatal regulation, antioxidant defense, and osmotic homeostasis under abiotic stress conditions. The co-occurrence of these cis-elements indicates a functional role for these genes in mediating ABA- and methyl jasmonate (MeJA)-dependent stress response pathways. Notably, the TGA-element and ABRE were especially prominent among the developmentally associated regulatory elements, suggesting conserved hormonal regulation functions.

Moreover, elements involved in responses to environmental stress, particularly the anaerobic responsive element (ARE), were frequently detected. Cis-elements related to developmental processes, such as those associated with zein metabolism, CAT-box, and the GCN4-motif, were also identified, indicating a possible role for *ERECTA*, *TMM*, and *SERK* genes in seed development and broader developmental regulation, consistent across both upland cotton species (Fig. S8). Clustering and enrichment analyses of cis-elements revealed conserved regulatory convergence within gene families. Distinct element profiles were observed among the *ERECTA*, *TMM*, and *SERK* families: *ERECTA* promoters contained predominantly light-responsive motifs (3–7 Box 4 motifs and 1–3 TCT-motifs per promoter); *TMM* promoters were enriched in hormone-responsive elements (typically two CGTCA/TGACG motifs per promoter); and *SERK* promoters featured a high abundance of light-responsive elements (2–8 Box 4 motifs), with approximately 20% containing 2–5 JA-responsive motifs and about 60% harboring multiple AREs. Collectively, these results highlight the conserved yet complex cis-regulatory landscape of *ERECTA*, *TMM*, and *SERK* gene families, underscoring their multifaceted roles in coordinating environmental stress responses and developmental signaling in cotton.

### Prediction of microRNA-mediated regulation of *ERECTA*,* TMM*, and *SERK* genes based on interaction networks

**Fig. 6:**
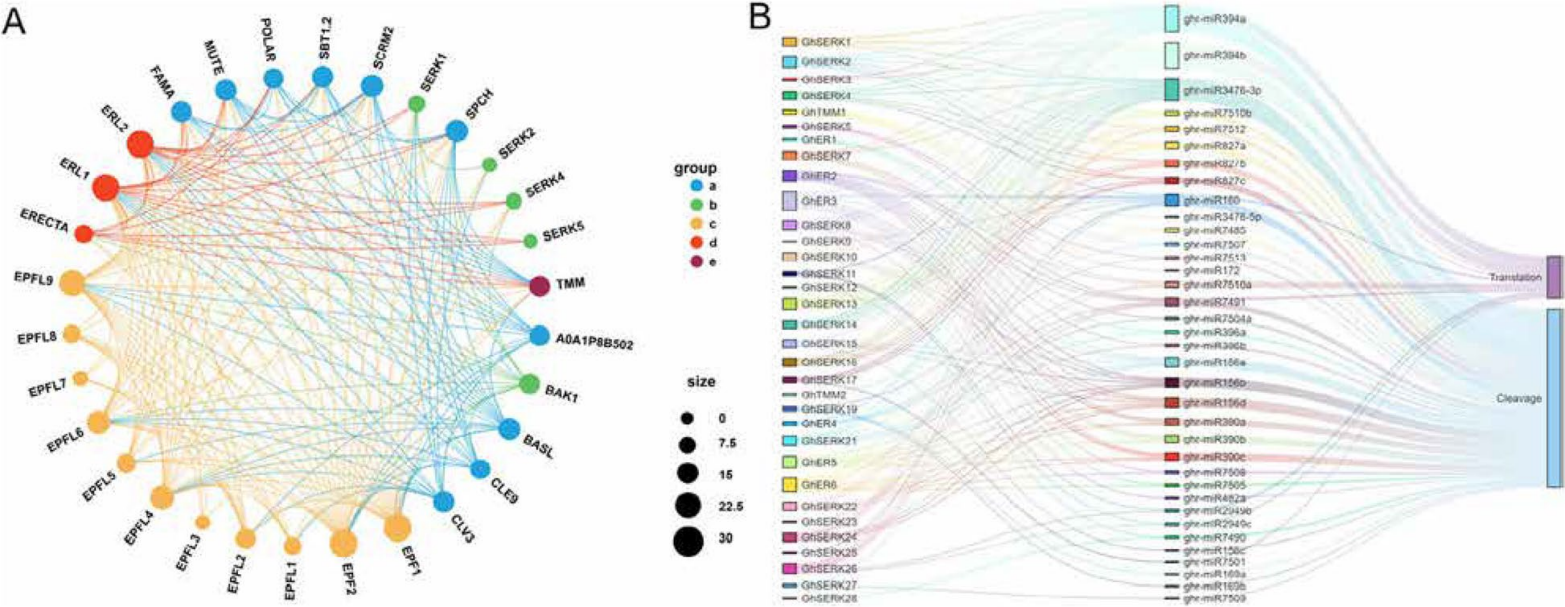
Regulatory relationship and interaction network diagram. **A** Protein–protein interaction analysis of EPF, ER, and TMM genes based on the STRING database; (**B**) Interaction network between predicted miRNAs and their target genes (*GhERECTA*, *GhTMM*, and *GhSERK*) generated using CNSknet software. Rectangles in the center represent miRNAs, while those on the left represent *GhERECTA*, *GhTMM*, and *GhSERK* genes

To elucidate the functional landscape of EPF/EPFL proteins in upland cotton (*Gossypium hirsutum*), a protein–protein interaction (PPI) network was constructed to investigate potential regulatory relationships among members of the *ERECTA*, *TMM*, and *SERK* gene families. Due to the limited availability of experimentally validated interaction data for cotton, we employed interaction data from *Arabidopsis thaliana* as a reference framework, incorporating both experimentally supported and predicted interactions. Orthologous relationships between *Arabidopsis* proteins and their *G. hirsutum* counterparts were established based on high sequence identity (≥ 60%), as detailed in Supplementary Table 8. Using a homology-based approach implemented via the STRING database, we predicted a robust network of interactions among EPF/EPFL ligands and their corresponding *ERECTA*, *TMM*, and *SERK* receptors, consistent with their established roles in ligand–receptor-mediated signaling (Fig. 6A). Furthermore, the network revealed potential crosstalk between these receptor kinase modules and other signaling components, including CLE/CLV peptides and bHLH transcription factors such as FAMA and SCRM2, suggesting broader functional integration of these pathways in the regulation of stomatal development.

MicroRNA (miRNA) regulation of these target genes was further analyzed. Among the 36 miRNAs predicted to regulate *ERECTA*, *TMM*, and *SERK* genes, *ghr-miR3476-3p* and *ghr-miR3476-3p* showed broad regulatory potential, targeting up to 19 receptor genes. Conversely, *ghr-miR156c*, *ghr-miR169a/b*, and *ghr-miR172* exhibited specificity toward individual genes (Fig. 6B). Notably, *ghr-miR3476-3p* demonstrated the broadest regulation across receptor genes, highlighting its potential role as a central regulator. Moreover, the strong specificity observed for miRNAs like *ghr-miR156c* and *ghr-miR172* suggests precise regulatory roles. These findings indicate that miRNA-mediated regulation of *ERECTA*, *TMM*, and *SERK* gene expression is finely tuned and potentially critical for modulating developmental plasticity and stress adaptation in cotton.

### Tissue-specific expression patterns of *EPF/EPFL*, *ERECTA*, *TMM*, and *SERK* Genes

**Fig. 7:**
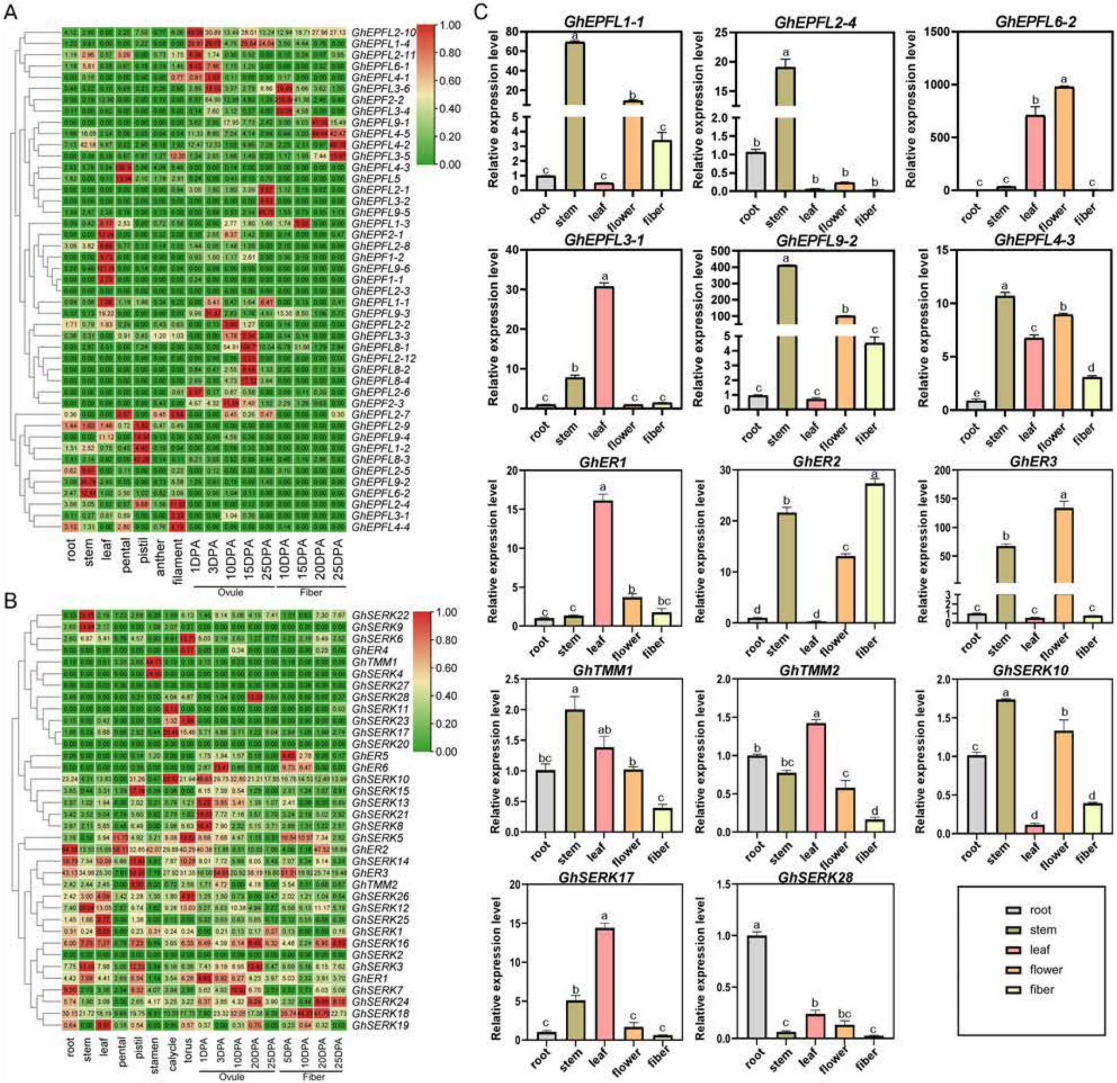
Expression patterns of *GhEPF/EPFL*, *GhERECTA*, *GhTMM*, and *GhSERK* genes in different tissues (**A**) Tissue-specific expression patterns of GhEPF/EPFL genes in root, stem, leaf, petal, pistil, anther, filament, ovule, and fiber; (**B**) Tissue-specific expression patterns of *GhERECTA*, *GhTMM*, and *GhSERK* genes in root, stem, leaf, petal, pistil, stamen, calycle, torus, ovule, and fiber;. **C** Expression profiles of selected *GhERECTA*, *GhTMM*, and *GhSERK* genes across different tissues. Error bars represent standard deviations (SD) based on three independent biological replicates (*n* = 3). Statistical differences among tissues were evaluated using one-way ANOVA, and significance is indicated by different lowercase letters (*P* ≤ 0.05). Groups sharing the same letter are not significantly different

Tissue-specific expression analysis of *GhEPF/EPFL* (45 genes), *GhERECTA* (6 genes), *GhTMM* (2 genes), and *GhSERK* (28 genes) in *G. hirsutum* revealed distinct regulatory patterns. *GhEPF/EPFL*s were broadly expressed in root, stem, leaf, petal, pistil, stamen, and ovule tissues but exhibited clear tissue-specific preferences related to developmental stages (Fig. 7A). Specifically, *GhEPFL9-6* showed leaf-specific expression, *GhEPFL4-3* was highly enriched in petals, and *GhEPFL8-1*, *GhEPF2-2*, *GhEPFL9-5*, and *GhEPFL4-2* exhibited stage-specific activation during ovule (1–20 DPA) and fiber (5–25 DPA) development. Expression in root tissues was generally low, whereas stem tissues exhibited notable expression of *GhEPFL4-2* and *GhEPFL9-2* (Fig. 7A).

Among the receptor genes, *GhER1*-*3* exhibited significantly higher expression levels across various tissues and developmental stages compared to *GhER4*-*6*. *GhTMM1*-*2* generally demonstrated low expression levels overall, though tissue-specific high expression was observed in particular tissues. The *GhSERK3*, *5*, *10*, *12*, *16*, *18*, and 24 genes displayed relatively higher expression patterns throughout the entire plant when compared to other *SERK* family members. Additionally, certain *SERK* genes showed tissue-restricted expression profiles: *SERK2* and *SERK9* exhibited elevated expression in stems, while *SERK4* expression was exclusively detected in stamens. Notably, *SERK2*, *SERK20*, and *SERK27* demonstrated no detectable expression across all examined tissues (Fig. 7B).

To validate the RNA-seq results, qRT-PCR analyses were conducted on selected *GhEPF/EPFL*, *ER*, *TMM*, and *SERK* genes with higher expression levels (Fig. 7C). Tissue-specific expression profiling revealed distinct spatial patterns: *GhEPFL1-1*, *GhEPFL2-4*, *GhEPFL4-3*, *GhEPFL9-2*, *GhTMM1*, and *GhSERK10* exhibited predominant expression in stem tissues, whereas *GhEPFL6-2*, *GhEPFL3-1*, *GhER1*, *GhTMM2*, and *GhSERK17* were enriched specifically in leaves. *GhER2* showed elevated expression primarily in floral tissues, and *GhSERK28* was predominantly expressed in roots.

### Differential expression of *EPF/EPFL*, *ERECTA*, *TMM*, and *SERK* genes under salt and drought stress in cotton

**Fig. 8:**
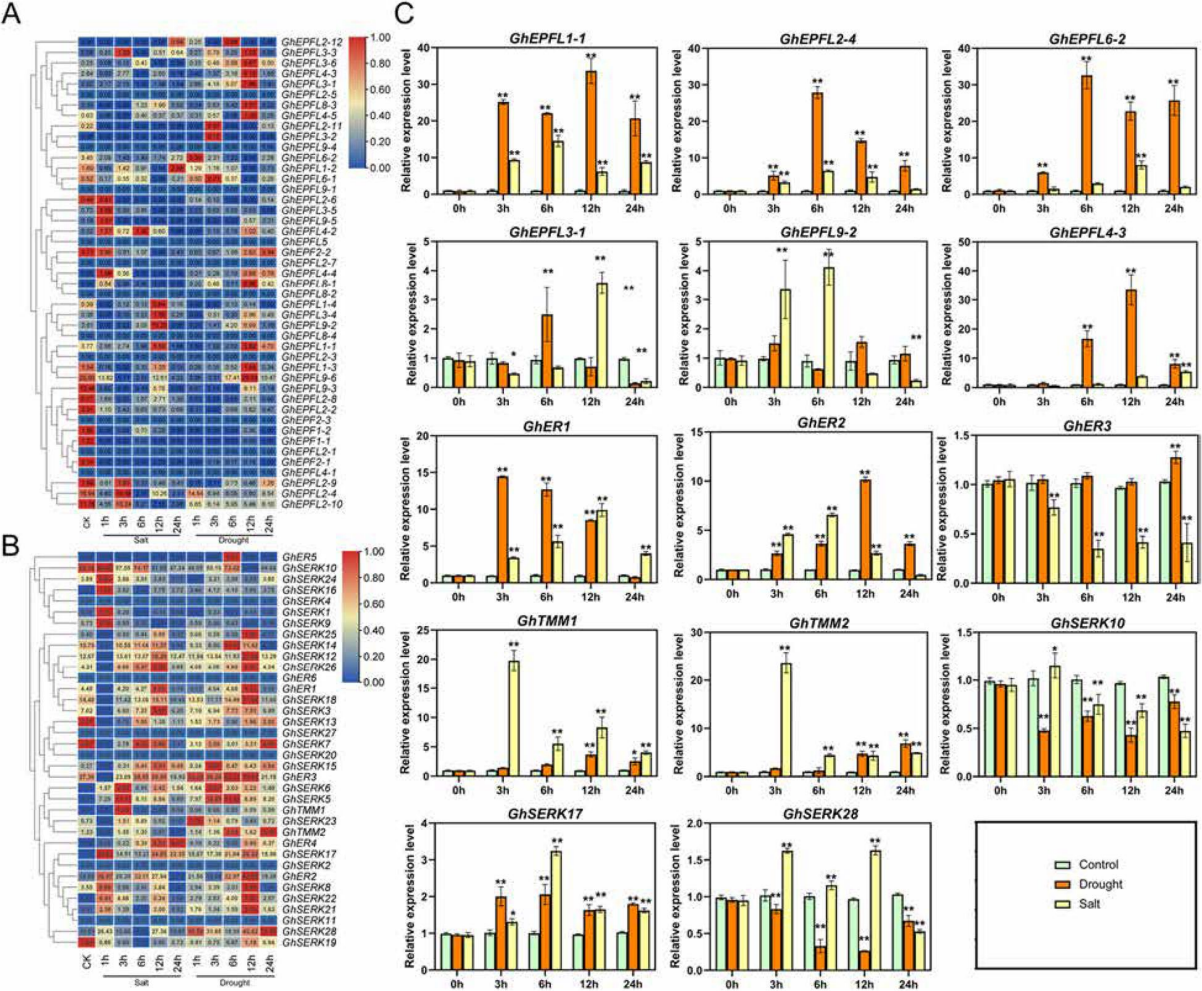
Expression profiles of selected *GhERECTA*, *TMM*, and *SERK* genes under drought and salt treatments. A Relative expression profiles of *GhEPF/EPFL* genes under drought and salt treatments. **B** Relative expression profiles of *GhERECTA*, *GhTMM*, and *GhSERK* genes under drought and salt treatments. **C** Expression profiles of selected *GhERECTA*, *TMM*, and *SERK* genes under drought and salt treatments.Error bars represent standard deviations (SD; *n* = 3) from independent biological replicates. Significant differences were determined by one-way ANOVA and are indicated by different lowercase letters (**P ≤ 0.01, *P ≤ 0.05)

Under salt and drought stress, *GhEPF/EPFL* genes exhibited dynamic, time-dependent induction patterns. *GhEPFL9-6*, *GhEPFL1-1*, and *GhEPFL2-10* showed constitutive expression across all tested stress conditions, reaching peak levels at 6–12 h post-treatment. Under drought stress, *GhEPFL3-6*, *GhEPFL3-3*, *GhEPF2-2*, and *GhEPFL4-2* were rapidly upregulated (1–12 h), with *GhEPF2-2* reaching peak expression at 24 h (Fig. 8A). Among receptor genes, *GhER1-3* and *GhTMM2* exhibited significant responsiveness to drought stresses, whereas *GhER3-6* and *GhTMM1* remained transcriptionally inactive. Within the *GhSERK* gene family, *GhSERK14*, *GhSERK10*, *GhSERK28*, *GhSERK12*, and *GhSERK18* displayed sustained induction in response to drought stress, while *GhSERK27*, *GhSERK20*, and *GhSERK11* showed no significant changes under tested stress conditions (Fig. 8B). Collectively, these findings elucidate a hierarchical regulatory network in which EPF/EPFL ligands and their associated ERECTA, SERK, and TMM receptors coordinate developmental processes and stress adaptation through precise spatiotemporal regulation.

To validate stress-responsive gene expression, we selected representative candidates from the *EPF/EPFL*, *ERECTA*, and *SERK* families based on their significant upregulation under drought conditions and relatively high transcript abundance. qRT-PCR analysis revealed that, under drought stress, *GhEPFL6-2*, *GhEPFL1-1*, *GhEPFL2-4*, *GhEPFL4-3*, *GhER1*, *GhER2*, and *GhSERK17* exhibited progressive induction at 3, 6, 12, and 24 h post-treatment, indicating a time-dependent activation of key components within the EPF–ERECTA signaling pathway. In contrast, salt stress specifically upregulated *GhEPFL9-2*, *GhTMM1*, *GhTMM2*, and *GhSERK28*, whereas *GhSERK10* showed consistent downregulation under both drought and salt conditions. Notably, *GhEPFL3-1* displayed transient induction in response to drought (at 6 h) and salt stress (at 12 h), while *GhER3* was transcriptionally repressed under salt stress but remained relatively stable under drought. Importantly, the qRT-PCR results were highly consistent with RNA-seq expression profiles, reinforcing the reliability of the observed transcriptional trends. Collectively, these findings underscore the dynamic, gene-specific, and stress-dependent regulatory responses of *ERECTA* signaling components in cotton, and further support the involvement of ABA- and JA-associated pathways in mediating these responses.

### VIGS of *GhEPFL1-1*, *GhER1*, and* GhSERK17* impairs drought tolerance by altering stomatal development and oxidative stress responses in cotton

**Fig. 9:**
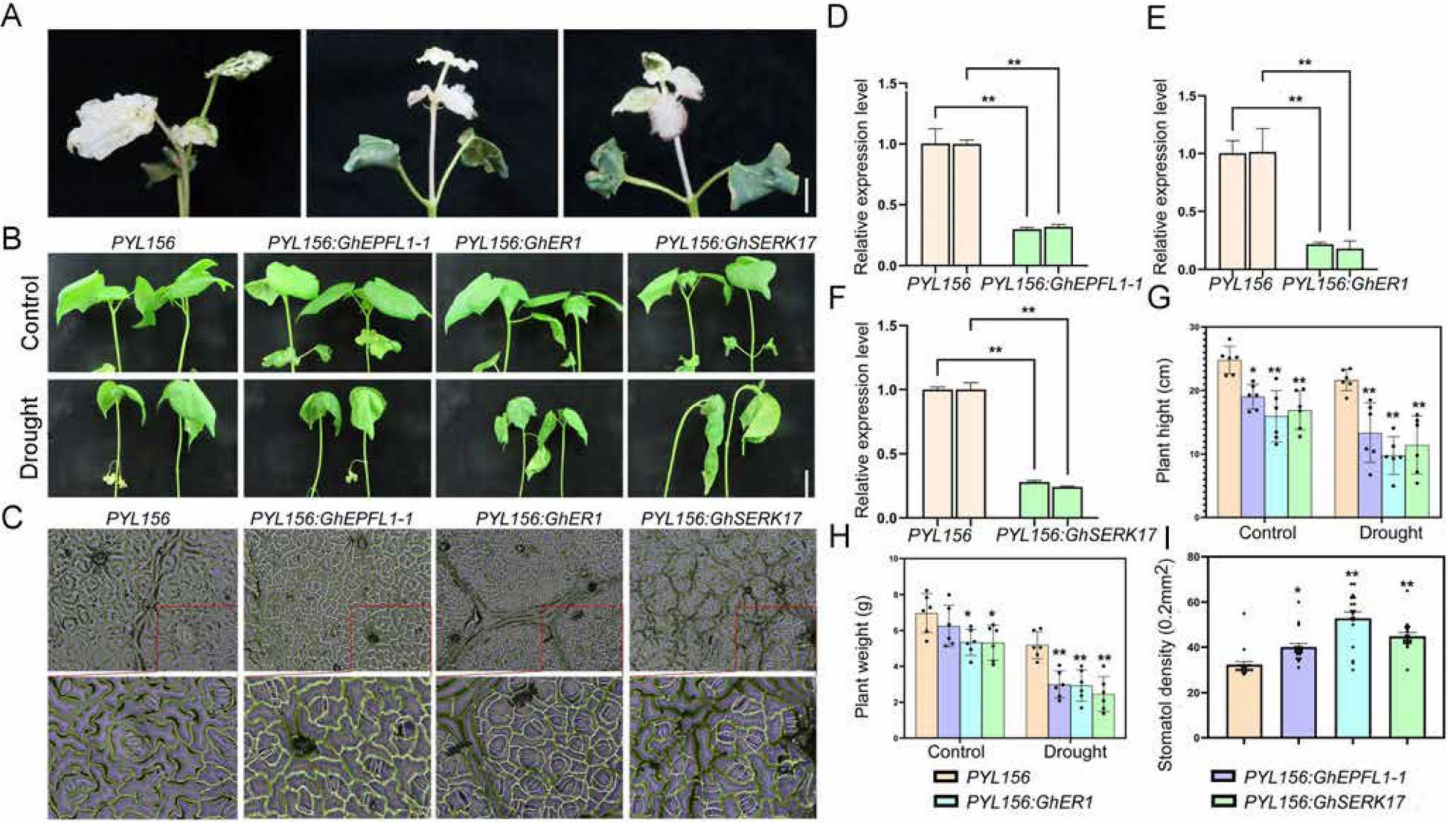
Silencing of *GhEPFL1-1*, *GhER1*, and *GhSERK1* reduces cotton tolerance to drought stress. **A** Albino phenotype observed in *PYL156*:*CLA1* lines; (**B**) Seedlings of *PYL156*:*GhEPFL1-1*, *PYL156*:*GhER1*, and *PYL156*:*GhSERK1* exhibited reduced drought tolerance compared to *PYL156*:*00* control seedlings. Scale bar = 2 cm; (**C**) Stomatal phenotype on the abaxial (lower) epidermis.Scale bar = 2 cm; (**D**–**F**) The analysis of *GhEPFL1-1*, *GhER1*, and *GhSERK1* expression levels in *PYL156:00* and VIGS-treated seedlings; (**G**–**I**) Physiological indices of drought response, including plant height, plant weight, and stomatal density. Error bars represent standard deviation (n = 3). Results are based on three independent experiments. Significant differences were determined by one-way ANOVA and are indicated by different lowercase letters (**P ≤ 0.01, *P ≤ 0.05)

To investigate the functional roles of GhEPFL1-1, GhER1, and GhSERK17 under drought stress, virus-induced gene silencing (VIGS) was performed in cotton plants. Gene silencing efficiency was confirmed by PCR (Fig. 9D–F), and the silenced plants exhibited a characteristic leaf-whitening phenotype (Fig. 9A). Notably, no significant differences in silencing efficiency were observed among the target genes under identical treatment conditions (Supplementary Table 9). Under drought conditions, silenced plants showed significantly increased wilting symptoms compared to the *PYL156* control (Fig. 9B). Notably, stomatal density on the abaxial leaf surface was markedly higher in the silenced lines relative to the controls (Fig. 9C, L), with the most pronounced increase observed in *GhER1*-silenced plants. Physiological assessments revealed impaired drought tolerance in silenced lines, indicated by slower growth rates and increased damage under drought stress (Fig. 9G, H). These results support the hypothesis that *GhEPFL1-1*, *GhER1*, and *GhSERK17* negatively regulate stomatal development, and their downregulation disrupts drought-adaptive mechanisms, consistent with their roles within the *ERECTA* signaling pathway.

**Fig. 10:**
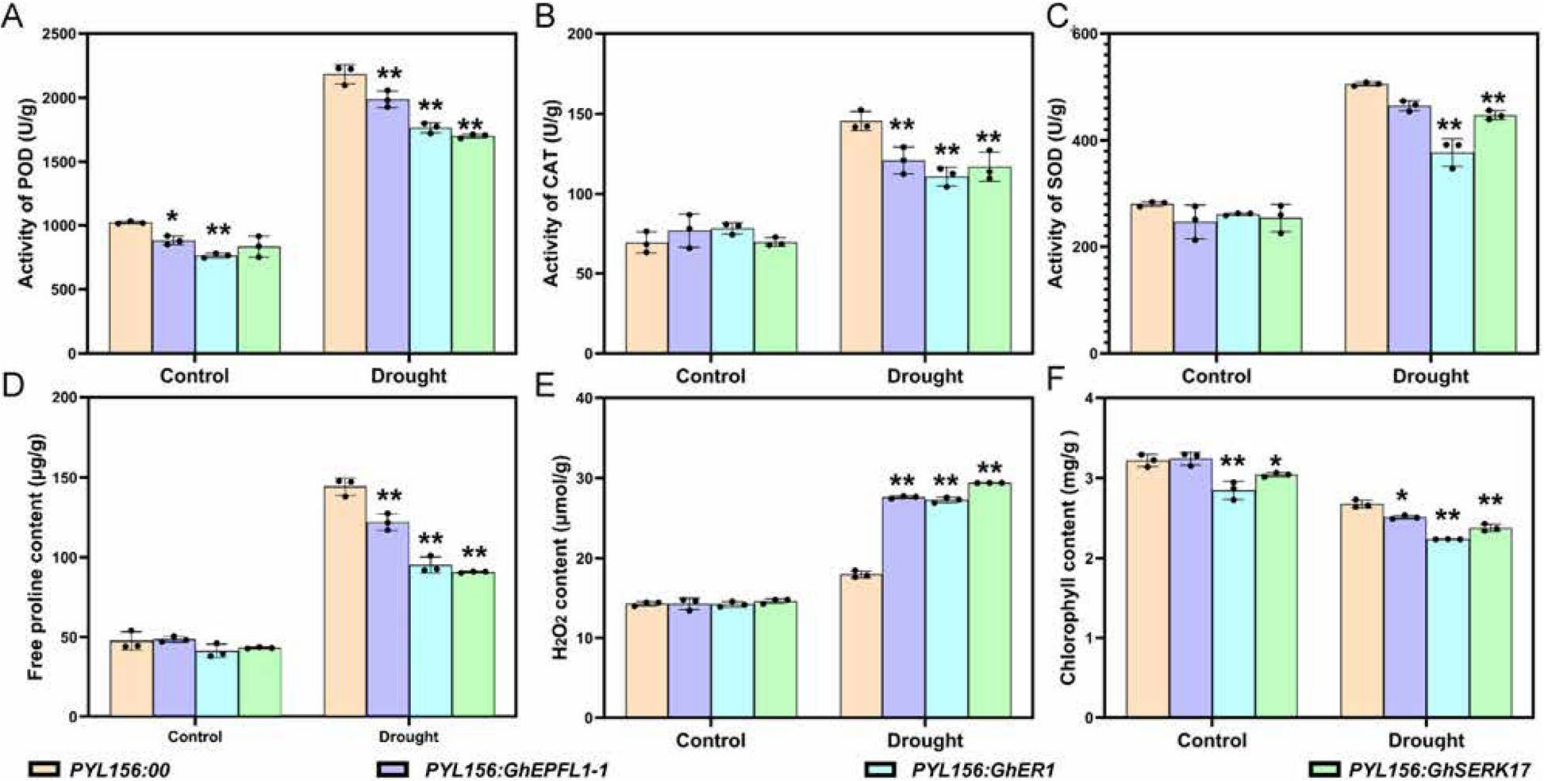
Silencing of *GhEPFL1-1*, *GhER1*, and *GhSERK1* Reduces Drought Tolerance in *G. hirsutum*. **A** Peroxidase (POD) activity; (**B**) Catalase (CAT) activity; (**C**) Superoxide dismutase (SOD) activity; (**D**) Free proline content; (**E**) Hydrogen peroxide (H₂O₂) content; (**F**) Chlorophyll content. Results are presented as the means of three independent experiments. Significant differences are indicated by different lowercase letters (**P ≤ 0.01, *P ≤ 0.05), as determined by one-way ANOVA

To further investigate the physiological consequences of silencing *GhEPFL1-1*, *GhER1*, and *GhSERK17* under drought stress, key physiological parameters were evaluated. Under normal conditions, total superoxide dismutase (SOD) activity did not significantly differ between the silenced lines (*PYL156*:*GhEPFL1-1*, *GhER1*, *GhSERK17*) and the control. However, drought stress resulted in significantly reduced SOD activity in the silenced plants compared to *PYL156* (Fig. 10C). Similarly, peroxidase (POD) activity and proline (Pro) content exhibited notable differences: under normal conditions, POD activity was lower in *GhER1*-silenced plants compared to the control; under drought stress, silenced lines exhibited significantly lower POD activity and Pro content than *PYL156*, although still higher than the unstressed controls (Fig. 10A).

**Fig. 11:**
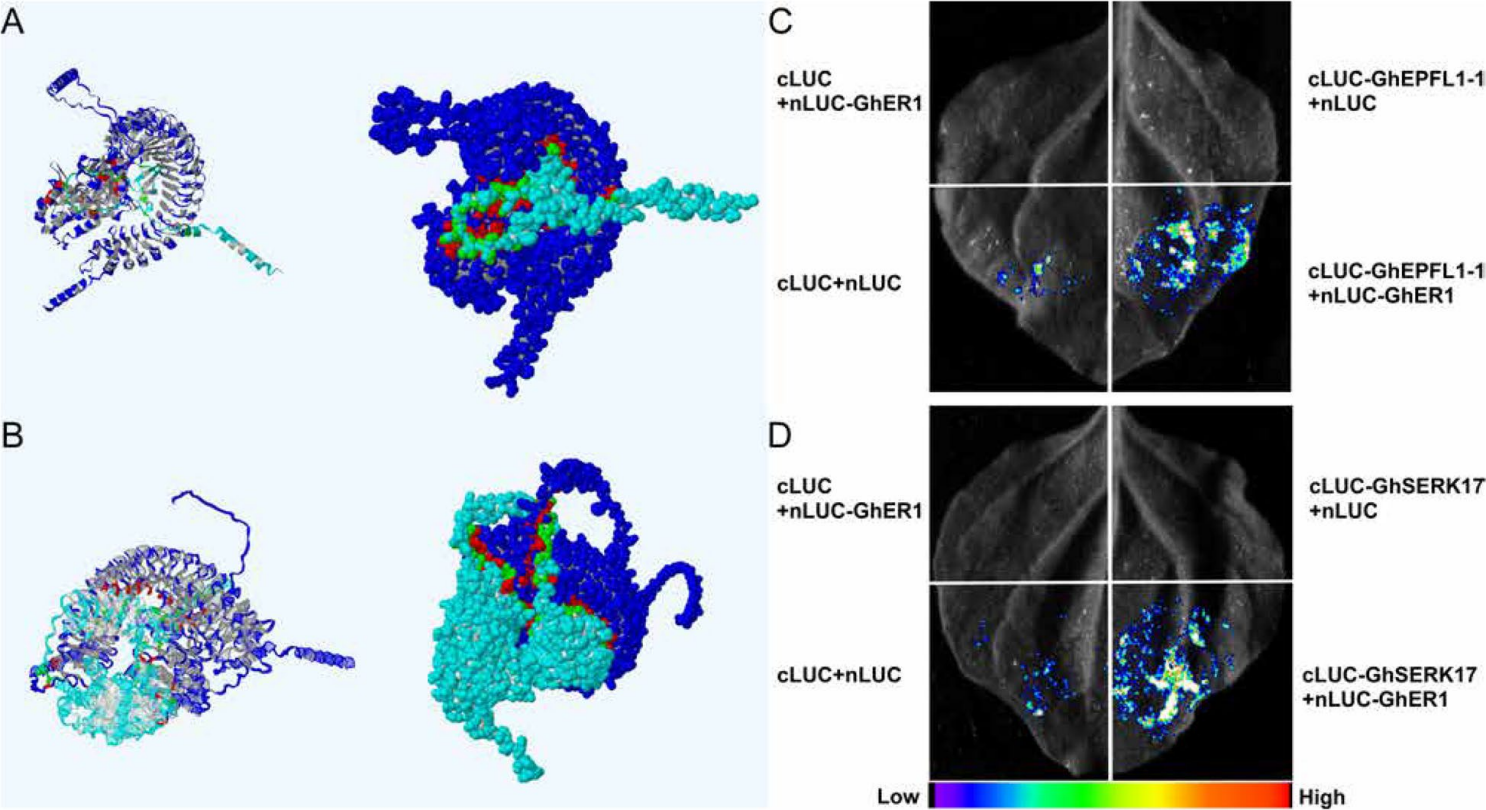
Split luciferase complementation assay reveals the interactions between GhEPFL1-1 and GhER1, and between GhSERK17 and GhER1. **A** Molecular model of the interaction between GhEPFL1-1 and GhER1 proteins. **B** Molecular model of the interaction between GhSERK17 and GhER1 proteins. **C** Split firefly luciferase complementation assay in *N. benthamiana* leaves showing the interaction between GhEPFL1-1 and GhER1. **D** Split firefly luciferase complementation assay in *N. benthamiana* leaves showing the interaction between GhSERK17 and GhER1

Moreover, oxidative stress indicators, including hydrogen peroxide (H_2_O_2_) and malondialdehyde (MDA), were significantly elevated in silenced plants under drought stress compared to *PYL156* controls, whereas no significant differences were found under normal conditions (Fig. 10B and D). Chlorophyll content also markedly decreased in silenced plants after drought treatment (Fig. 10E). Together, these findings demonstrate that silencing *GhEPFL1-1*, *GhER1*, and *GhSERK17* impairs cotton drought tolerance by disrupting physiological and biochemical stress-adaptive mechanisms, specifically through alterations in stomatal development and oxidative stress responses.

#### Molecular docking and experimental validation of GhEPFL1-1 Interaction with GhER1 and the Co-receptor GhSERK17 in cotton

To gain molecular insight into the potential ligand–receptor interactions among *GhEPFL1-1*, *GhER1*, and the co-receptor *GhSERK17*, molecular docking simulations were performed. Structural alignment analysis showed that both *GhEPFL1-1* (RMSD = 0.001; 932 atoms aligned) and *GhSERK17* (RMSD = 0.001; 4,592 atoms aligned) exhibited exceptionally high structural congruence with *GhER1*. Docking results revealed well-defined binding interfaces between the proteins, primarily mediated by conserved amino acid residues and critical structural domains (Figs. 10A, B; Supplementary Table 10). These computational predictions support a specific ligand–receptor recognition mechanism involving *GhER1* as the receptor and *GhSERK17* as its co-receptor. To experimentally validate these in silico findings, luciferase complementation imaging (LCI) assays were conducted in *Nicotiana benthamiana* leaves (Fig. 10C, D). *GhEPFL1-1* and *GhSERK17* were individually fused to the C-terminal fragment of luciferase (cLUC), while *GhER1* was fused to the N-terminal fragment (nLUC). Co-expression of nLUC-*GhEPFL1-1* with cLUC-*GhER1*, or nLUC-*GhER1* with cLUC-*GhSERK17*, produced strong luminescence signals, confirming direct and specific interactions among these proteins. In contrast, negative controls expressing only individual nLUC or cLUC constructs did not produce detectable luminescence, reinforcing the specificity of the observed interactions. Collectively, these molecular docking and LCI assay results demonstrate that *GhEPFL1-1* specifically interacts with *GhER1* and its co-receptor *GhSERK17*, supporting their coordinated roles in regulating stomatal development and drought stress responses in cotton.

## Discussion

The EPF/EPFL family comprises cysteine-rich secreted peptides critical for intercellular and interorgan communication in plants. These peptides function as long-distance signaling molecules regulating key physiological processes, including plant growth, development, and stress responses [11, 18, 31, 52, 60]. Stomata represent the primary conduits for water loss through transpiration; hence, optimizing stomatal density is essential for enhancing water-use efficiency and drought tolerance. Previous studies have demonstrated improved drought resilience in monocots such as rice (*Oryza sativa*), maize (*Zea mays*), and wheat (*Triticum aestivum*) through manipulation of stomatal density [6, 10, 42]. Similarly, EPF/EPFL-mediated stomatal regulation has been extensively documented in dicots, including *A. thaliana*, tomato (*Solanum lycopersicum*), poplar (*Populus trichocarpa*), and potato (*Solanum tuberosum*) [28, 37]. In *Arabidopsis*, disruptions in *EPFL* (*EPFL1*, *EPFL2*, *EPFL4*, *EPFL6*) or ER-family receptor genes (*ER*, *ERL1*, *ERL2*) compromise epidermal patterning and ovule development [45–47], while EPF specifically interacts with ER-family receptors to regulate leaf margin morphogenesis [31, 33, 34]. Our study provides new evidence that GhEPFL1-1 directly interacts with GhER1, which further forms a functional co-receptor complex with GhSERK17 in upland cotton (*G. hirsutum*).

Co-receptor complexes in plants commonly involve ERECTA (ER) receptors interacting with other receptor-like kinases, such as TMM and SERKs. These complexes perceive extracellular EPF/EPFL ligand signals and initiate intracellular signaling cascades through receptor transphosphorylation events [34]. In the present study, we systematically identified receptor candidates of cotton EPF peptides through genome-wide analyses of ERECTA, TMM, and SERK families. Our comparative genomic analysis indicated that diploid cotton species (*G. arboreum* and *G. raimondii*) possess similar gene copy numbers for ERECTA (3 genes) and TMM (1 gene) as *Arabidopsis*, while allotetraploid cotton species (*G. hirsutum* and *G. barbadense*) exhibit duplicated gene sets (6 *ERECTA* and 2 *TMM* genes), likely arising from whole-genome duplication events during polyploidization. A comparable expansion was also evident in the *SERK* family, where tetraploid cotton species harbored significantly more *SERK* genes (28–30 genes) than diploid cottons (16 genes), markedly exceeding *Arabidopsis* (5 genes). This expansion pattern suggests that polyploidization and subsequent lineage-specific gene duplication events significantly contributed to the diversification and potential functional specialization of the SERK family during cotton evolution.

Phylogenetic analysis revealed that the *ERECTA*, *TMM*, and *SERK* gene families clustered into six distinct subclades (Fig. 1A). The *SERK* family was further subdivided into three well-supported subgroups, while all *ERECTA* genes formed a single, independent clade. In the *TMM* family, cotton homologs clustered together into one lineage, whereas *Arabidopsis TMM* genes formed a separate group. These patterns indicate lineage-specific expansion and divergence, particularly within the *SERK* family in *Gossypium*. Members of closely related *SERK* subgroups exhibited conserved exon–intron structures and similar protein domain architectures (Fig. 2C), suggesting potential functional conservation within each clade. Within the *ERECTA* lineage, structural divergence was observed between two branches: one group featured an additional exon and distinct motif compositions compared to the other (Fig. 2B). For the *TMM* family, phylogenetic divergence between *Arabidopsis* and cotton was primarily associated with variations in C-terminal conserved motifs (Fig. 2A). These interspecies structural differences are consistent with the phylogenetic groupings and support the hypothesis that subgenome-specific evolutionary trajectories have contributed to functional diversification. The observed concordance between evolutionary relationships and structural features underscores the combined influence of gene duplication events and sequence divergence in shaping the evolution of receptor-like kinase gene families in cotton.

Gene structure analysis revealed high conservation in the exon–intron organization of ERECTA, TMM, and SERK family members across cotton species. Cotton *ERECTA* genes typically contain two exons and 27 introns, mirroring the structural organization observed in *Arabidopsis ER* genes. This structural similarity strongly implies functional conservation within the *ERECTA* gene family. Conserved protein motifs across cotton ERECTA members further support this hypothesis, although the presence of divergent motifs in certain family members suggests functional diversification. The cotton *TMM* gene family exhibited nearly identical exon–intron structures, further underscoring their evolutionary conservation. Similarly, most cotton *SERK* genes consistently comprised two exons and 11 introns, a conserved structural pattern widely observed in *Arabidopsis*, Paulownia, rice (*Oryza sativa*), apple (*Malus domestica*), and moso bamboo (*Phyllostachys edulis*) [34, 49, 63–65].

Cis-acting element analysis indicated that the promoters of cotton *ER*, *TMM*, and *SERK* genes potentially regulate diverse biological processes, including transcriptional regulation, developmental control, hormone signaling (e.g., methyl jasmonate [MeJA], abscisic acid [ABA], auxin [IAA]), and responses to biotic and abiotic stresses (Fig. 5A, B). Consistent with these predictions, gene expression analyses in *G. hirsutum* revealed substantial variation across different tissues, exhibiting ubiquitous, tissue-specific, or low-level expression patterns, indicative of functional specialization or redundancy. Under abiotic stresses, these genes exhibited distinct transcriptional dynamics, aligning well with previous reports of *EPF*, *SERK*, and *ER* gene responsiveness to environmental stresses such as heat, salinity, and drought [11, 18, 52].

The EPF-ER signaling pathway plays a pivotal role in plant stress responses by regulating stress-tolerance genes through receptor-ligand interactions and downstream transcriptional regulation. Our functional validation via VIGS of *GhEPFL1-1*, *GhER1*, and *GhSERK17* in cotton demonstrated that these genes modulate stomatal density, significantly affecting drought resistance. Notably, silenced plants displayed increased stomatal density, decreased chlorophyll content, reduced antioxidant enzyme activities, and elevated oxidative stress markers compared to controls (Figs. 9 and 10). These observations indicate that GhER1 and its co-receptor GhSERK17, mediated by GhEPFL1-1 ligand interaction, play a crucial role in coordinating stomatal development and drought-stress adaptation mechanisms. Luciferase complementation imaging assays further confirmed the direct interactions between GhEPFL1-1 and GhER1, and the critical involvement of GhSERK17 as a co-receptor in forming functional receptor complexes. These findings collectively underscore the functional conservation and adaptive diversification of the EPF/EPFL-ER signaling network in stomatal regulation and stress responses, providing critical insights for improving drought tolerance and water-use efficiency in cotton breeding. The dual roles of GhEPFL1-1, GhER1, and GhSERK17 in coordinating developmental processes and stress adaptation warrant thorough investigation as potential breeding targets. These genes regulate stomatal development and patterning, offering a molecular lever to optimize transpiration efficiency and drought resistance by reducing stomatal density under water-limited conditions (Fig. 11).

The selection of *Gossypium hirsutum* TM-1 for stress treatments and functional validation was based on its status as a widely used genomic reference line, with well-characterized drought-responsive traits and a fully sequenced genome. Its comprehensive transcriptomic resources also facilitate reliable cross-species comparisons with model plants such as *Arabidopsis thaliana*, enabling mechanistic insights to be contextualized within a defined genomic framework. However, we acknowledge that restricting validation to TM-1 may limit generalizability. Future studies involving multiple *Gossypium* cultivars will be essential to assess the broader applicability of these signaling mechanisms across genetic backgrounds.

## Conclusion

In this study, we systematically investigated the *EPF/EPFL* and receptor gene families (*ERECTA*, *TMM*, and *SERK*) across multiple cotton species, elucidating their genomic organization, evolutionary dynamics, and functional specialization. Through comprehensive genomic, structural, and expression analyses, we revealed that polyploidization and lineage-specific gene expansions significantly contributed to the diversification of these signaling components in cotton. Functional analyses confirmed that VIGS of GhEPFL1-1, GhER1, and the co-receptor GhSERK17 significantly increased stomatal density and reduced enzymatic antioxidant activities and chlorophyll content, ultimately impairing drought resistance in cotton. Furthermore, luciferase complementation imaging assays experimentally validated the direct ligand-receptor interactions, confirming that GhEPFL1-1 specifically binds GhER1 and forms a functional co-receptor complex with GhSERK17. These interactions regulate stomatal patterning and drought stress responses. Collectively, our findings provide a robust molecular framework for EPF/EPFL-mediated stomatal development and stress adaptation in cotton. The identified signaling components represent promising genetic targets for improving drought resilience and water-use efficiency in cotton breeding programs, underscoring their evolutionary conservation and broad applicability in crop improvement strategies.

## Supplementary Information

Supplementary Material 1.

Supplementary Material 2.

Supplementary Material 3.

Supplementary Material 4.

Supplementary Material 5.

Supplementary Material 6.

Supplementary Material 7.

Supplementary Material 8.

Supplementary Material 9.

Supplementary Material 10.

## Data Availability

All data generated or analyzed during this study are included in this published article and its supplementary information files. Additional datasets used and/or analyzed during the current study are available from the corresponding author on reasonable request.
